# Mild traumatic brain injury impairs the coordination of intrinsic and motor-related neural dynamics

**DOI:** 10.1016/j.nicl.2021.102841

**Published:** 2021-10-01

**Authors:** Lukas Rier, Rouzbeh Zamyadi, Jing Zhang, Zahra Emami, Zelekha A. Seedat, Sergiu Mocanu, Lauren E. Gascoyne, Christopher M. Allen, John W. Scadding, Paul L. Furlong, Gerard Gooding-Williams, Mark W. Woolrich, Nikos Evangelou, Matthew J. Brookes, Benjamin T. Dunkley

**Affiliations:** a Sir Peter Mansfield Imaging Centre, School of Physics and Astronomy, University of Nottingham, University Park, Nottingham NG7 2RD, UK; b Diagnostic Imaging, Hospital for Sick Children, Toronto, Canada; c Oxford Centre for Human Brain Activity, Warneford Hospital, University of Oxford, Oxford, UK; d Institute of Health and Neurodevelopment, Aston University, Birmingham, UK; e Mental Health and Clinical Neurosciences Academic Unit, School of Medicine, University of Nottingham, Queen’s Medical Centre, Nottingham, UK; f Neurosciences & Mental Health, Hospital for Sick Children Research Institute, Toronto, Canada; g Medical Imaging, University of Toronto, Toronto, Canada; h Faculty of Medicine, University of Toronto, Toronto, Canada; i National Hospital for Neurology and Neurosurgery, London, UK

**Keywords:** MEG, mild Traumatic Brain Injury, mTBI, Concussion, Networks, Beta bursts

## Abstract

Mild traumatic brain injury (mTBI) poses a considerable burden on healthcare systems. Whilst most patients recover quickly, a significant number suffer from sequelae that are not accompanied by measurable structural damage. Understanding the neural underpinnings of these debilitating effects and developing a means to detect injury, would address an important unmet clinical need. It could inform interventions and help predict prognosis. Magnetoencephalography (MEG) affords excellent sensitivity in probing neural function and presents significant promise for assessing mTBI, with abnormal neural oscillations being a potential specific biomarker. However, growing evidence suggests that neural dynamics are (at least in part) driven by transient, pan-spectral bursting and in this paper, we employ this model to investigate mTBI. We applied a Hidden Markov Model to MEG data recorded during resting state and a motor task and show that previous findings of diminished intrinsic beta amplitude in individuals with mTBI are largely due to the reduced beta band spectral content of bursts, and that diminished beta connectivity results from a loss in the temporal coincidence of burst states. In a motor task, mTBI results in diminished burst amplitude, altered modulation of burst probability during movement, and a loss in connectivity in motor networks. These results suggest that, mechanistically, mTBI disrupts the structural framework underlying neural synchrony, which impairs network function. Whilst the damage may be too subtle for structural imaging to see, the functional consequences are detectable and persist after injury. Our work shows that mTBI impairs the dynamic coordination of neural network activity and proposes a potent new method for understanding mTBI.

## Highlights

• MTBI is poorly understood and lacks objective diagnostic and prognostic tools.
• Abnormal neural oscillations are found in subjects with a history of mTBI.
• We identify transient bursts in MEG data using a Hidden Markov Model.
• We explain a deficit in beta connectivity and power in terms of transient bursts.
• Data-driven feature selection identifies symptom-relevant functional connections.

## 1 Introduction:

With an estimated 27 million annual cases worldwide, traumatic brain injury (TBI) causes a substantial burden on health services (James et al., 2019). Around 80% of cases are classified as mild traumatic brain injury (mTBI or concussion) (Cassidy et al., 2004), which is defined as the mostly transient impairment to mental function following a blow to the body (usually to the head, face or neck) (McCrory et al., 2017). The criteria distinguishing mTBI from moderate or severe types of brain injury typically include no or short-lived loss of consciousness (less than 30 min), post-traumatic amnesia (none or less than 24 h), and Glasgow Coma Scale of 13–15 on presentation to the emergency department (Carroll et al., 2004). Whilst most individuals with mTBI recover quickly and spontaneously, some continue to experience persistent symptoms after an mTBI, including difficulties with concentration, attention, memory, confusion, and slowness of thinking. These ‘mild’ symptoms can have a severe impact on quality of life. Despite the debilitating symptoms, mTBI, by definition, is not associated with demonstrable abnormalities on routine imaging with CT or MRI. Yet neurocognitive sequelae and symptoms suggest neural dysfunction persists after injury. Moreover, there are few predictors of outcome and treatment options are limited. Consequently, new objective means to understand the neuropathology of mTBI, to prognosticate, and inform intervention, are required.

Electrophysiological imaging provides a potent approach to understanding the functional consequence and impairment after mTBI. Magnetoencephalography (MEG) allows for sensitive functional imaging of neural activity and dynamics, by measuring the magnetic fields generated by neuronal current flow in the brain. Mathematical modelling of these fields yields 3D images of brain electrophysiology, with unmatched non-invasive insight into micro-, meso- and macroscopic neural circuits that dynamically form and dissolve to underpin cognition. The MEG signal is dominated by ‘neural oscillations’ (rhythmic electrical activity generated by neural assemblies) which are traditionally divided into canonical frequency bands (delta (1–4 Hz), theta (4–8 Hz), alpha (8–13 Hz) beta (13–30 Hz) and gamma (30 + Hz). Abnormalities in these signals are an indication of pathology, and several putative atypical oscillatory signatures of mTBI have been reported.

Much of the literature has focused on “pathological slowing” of rhythmic brain activity, including elevated low-frequency power, in particular the delta band. For example, Lewine and colleagues reported increased delta power in subjects with histories of mild head trauma (Lewine et al., 1999). Huang et al. (2014) reported abnormally high delta power in around 85% of subjects in a cohort of military veterans with mTBI, compared to healthy controls. Increased slow wave power has also been observed in cases of sports-related mTBI (Proskovec et al., 2020). These observations are consistent with animal studies where increased slow-wave activity was observed following the controlled induction of white-matter lesions (Gloor et al., 1977), suggesting abnormal delta is a consequence of white matter damage. In addition to slow-wave abnormalities, recent work suggests high-frequency dysfunction: for example, Huang and colleagues reported abnormal resting-state gamma activity in subjects with combat-related mTBI (Huang et al., 2020) and Zhang et al. demonstrated a decrease in beta amplitude in mTBI patients (Zhang et al., 2020). These high-frequency fingerprints are driven by distinct neurophysiological processes and open new routes to understanding the pathology of mTBI.

The heterogeneity of sequelae of mTBI is thought to be driven by diffuse yet subtle white matter damage, particularly around the corpus callosum (Aoki et al., 2012), affecting communication between distal, functionally specific regions. The degree of communication between regions can be tested directly using MEG functional connectivity assessment. Functional connectivity is defined as the statistical interdependency between functional signals from spatially separate regions; for MEG this tends to mean assessment of either coherence, phase synchrony, or amplitude envelope correlation, between oscillations in frequency bands of interest (O’Neill et al., 2015a). The beta band is of particular interest since it appears crucial in the establishment of canonical resting-state networks (Brookes et al., 2011, Hipp et al., 2012) as well as the dynamic orchestration of neural activity (Little et al., 2019, Sherman et al., 2016). Given the presumed diffuse nature of the disruptions caused by mTBI, the beta band is, therefore, a good candidate to assess this brain injury. In support of this, Zhang et al. showed that machine learning can classify injured states with high accuracy and that beta connectivity was an important feature for classification, showing that dysfunctional beta connectivity is an important marker of mTBI (Zhang et al., 2020).

All of the above assumes the “classical” view that oscillatory brain function is largely smoothly modulating rhythmic activity– this view is impoverished, as paradigm-shifting work now shows that transient, dynamic burst states are a fundamental mode of neural functioning (van Ede et al., 2018). “Oscillatory” power does not stem from oscillations *per se*, but rather it results, at least in part, from transient events of high amplitude activity, the spectral content of which intersects canonical frequency ranges, particularly beta band (Jones, 2016, Little et al., 2019, Sherman et al., 2016, Zich et al., 2020). When averaging across trials, these events sum to give the impression of slowly modulating oscillations - when in fact, for example, apparently sustained beta activity around motor events results from pan-spectral bursts whose probability of occurrence changes throughout the task (Little et al., 2019, Seedat et al., 2020).

This evolution in the understanding of neural dynamics shows that classical measures of oscillatory amplitude should also be supplemented with nascent metrics like “burst likelihood” and “burst duration.” This model also offers a novel means of calculating communication and connectivity. Seedat et al. used a Hidden Markov Model (HMM) to identify bursts and showed that the beta band connectome can be explained by the temporal coincidence of bursting (Seedat et al., 2020). Using a similar technique, Gascoyne et al. showed that previously reported beta abnormalities in schizophrenia can be explained using the burst model (Gascoyne et al., 2021). The neurobiological origins of the beta burst have been studied through neural modelling (Jones, 2016, Sherman et al., 2016), which could provide a deeper understanding of the origin of signal abnormalities within the cortex and suggests that important features of these transient neural signals can be lost through averaging data from many epochs or trials. The use of these methods in mTBI would reveal novel information about the neural impairment underlying mTBI sequelae.

In this paper, we analyse MEG recordings in 52 subjects (29 mTBI subjects and 23 healthy controls) - a subset (50 subjects) of which was previously presented by Zhang et al*.* - using a burst framework. Zhang and colleagues have already shown that there are intrinsic deficiencies in both spontaneous beta power and connectivity (Zhang et al., 2020). Here, using a HMM approach, we test the hypothesis that *the previous observation of abnormal beta activity can be explained by deficits in burst structure (specifically, low beta burst amplitude)*. In the same resting-state data we hypothesise that *abnormalities in connectivity can be explained by the lack of temporal coordination between bursts in spatially separate brain regions*. We further use a machine learning (ML) approach to determine the characteristic connections that are disrupted in mTBI and to evaluate the utility of burst connectivity measures in ML-based mTBI diagnosis. We also present a novel analysis of (unpublished) motor task data acquired in the same subjects to test a hypothesis of abnormal connectivity during the well-known post-movement beta rebound (PMBR) - a marker for interhemispheric recalibration of the motor system, and by extension, an index of corpus callosum integrity (Tewarie et al., 2019). Assuming that mTBI disrupts white matter integrity, particularly around the corpus callosum, we hypothesise that *the coincident bursts in the motor cortex which drive the measurable PMBR and connectivity will be in relative deficit in mTBI subjects*.

## 2 Methods

**Fig. 1:**
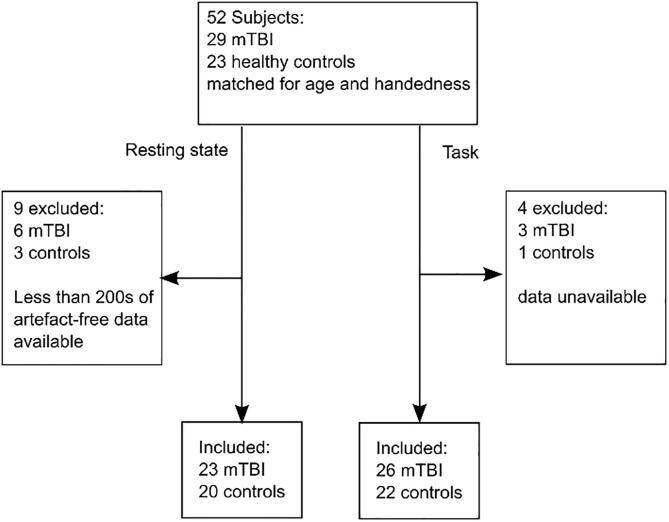
Patient enrolment flow chart.

In total 52 subjects were recruited to the study; 29 mTBI patients and 23 healthy controls. All subjects underwent two MEG recordings, resting state and a motor task. A flow chart showing subject inclusion is given in Fig. 1. Groups were matched for age, sex and handedness. All subjects gave written informed consent to take part in the study, which had been approved by the Research Ethics board of the Hospital for Sick Children, Toronto, Canada. All subjects in the mTBI group were scanned within 90 days of their injury (e.g. acute-subacute phase of injury; 39 ± 21 days (mean ± standard deviation (SD)), range: 7–88 days); mTBI subjects had undergone investigation by MRI (T1 weighted anatomical, detailed acquisition parameters in supplementary materials) and no positive clinical indications were found following review by a neuro-radiologist. In addition to MEG data acquisition, subjects were assessed for symptom severity using the Sport Concussion Assessment Tool 2 (SCAT2) on the day of scanning. The symptom evaluation contained in the SCAT2 battery of assessments asked subjects to subjectively rate the severity of 22 symptoms which span a range of clinical domains such as somatic (e.g. headache, neck pain), cognitive (e.g. feeling “in a fog”, difficulty concentrating), emotional and behavioural changes (e.g. more emotional, irritability), and sleep disturbance (e.g. drowsiness, Trouble falling asleep) (McCrory et al., 2009).

### 2.1 Paradigms

Data were recorded during two separate paradigms:

*Resting-state:* Subjects were asked to lie still, with their head in the MEG helmet, whilst 300 s of resting-state data were collected. Participants were in the supine position and were told to keep their eyes open. 23 individuals with mTBI (all male, aged 30 ± 7(mean ± SD)) were included in the study alongside 20 healthy controls (all male, aged 28 ± 5 (mean ± SD)). (These data were previously presented by (Zhang et al., 2020))
*Motor task:* We used a stimulus matching task with a 2-alternate forced-choice design that requires bilateral motor responses, previously described by Oh and colleagues (Oh et al., 2014). Subjects were simultaneously presented with three images – two stimuli on the upper left and right of a central fixation point and a third target image immediately below the fixation point. The subjects were asked to match one of the two stimuli presented in the upper visual field with the third target stimulus in the lower field, on either the colour or shape dimension, and indicate which of the stimuli matched the target by pressing a button with the corresponding left or right hand. Stimuli presentations lasted until a response was given (up to a maximum of 4 s) with a jittered inter-stimulus interval of 800–1200 ms. Recordings lasted for 370 trials. 26 individuals with mTBI (all male, aged 30 ± 7 years (mean ± SD)) were included in the study alongside 22 healthy controls (all male, aged 28 ± 5 (mean ± SD)). (These data have not been previously published.)

### 2.2 Data collection

All MEG data were collected using a 151-channel whole-head MEG system (CTF, Coquitlam, Vancouver, Canada) operating in a third-order gradiometer configuration, at a sampling frequency of 600 Hz. Before entering the scanner, three fiducial markers were placed on the subject’s head (left and right preauricular and nasion). These coils were energised to enable continuous tracking of the head position throughout the scan, and consequently, enable estimation of head movement.

### 2.3 Pre-processing

The MEG data were filtered using a 4th order Butterworth band-pass filter between 1 and 150 Hz and notch filters at 60 Hz (mains interference) and 120 Hz (harmonic). The continuous head motion information was used to define epochs in which the head position was maintained within 5 mm of its starting location, and head velocity was less than 5 mm/s; other epoch exclusion criteria were: SQUID resets in the MEG signal and/or signal discontinuities exceeding +/- 2pT. Each epoch was visually assessed for adequate quality by a trained MEG expert. Epochs not meeting these criteria were rejected. Independent component analysis (ICA) was applied to attenuate ocular (EOG) and cardiac (ECG) related artefacts. Identified components were removed manually following visual inspection.

For the Resting-state data, the post-ICA artefact-free recordings were segmented into 10 s epochs and only subjects with greater than 200 s (20 epochs) of clean data were included in the final analyses. The mean and standard deviation of available epochs for the resting state paradigm was 24.1 ± 0.6 for the mTBI group and 23.9 ± 0.6 for the control group.

The task data were epoched into 6 s segments; 3 s before and 3 s after a button press. Trials containing artefacts, or incorrect responses were excluded and only set-shift trials were used. For the task, an average (mean ± SD) of 175 ± 49 and 172 ± 33 epochs was included for the mTBI and control group respectively. For the mTBI group, this included 88 ± 29 left button presses and 88 ± 23 right button presses. For the control group, there were 87 ± 21 left button presses and 85 ± 19 right button presses. There were no significant differences in trial count between groups as assessed by a 2-sided T-test. Note also that reaction time did not differ significantly between groups (For left button presses, reaction times were 0.50 ± 0.12 s for controls and 0.55 ± 0.08 for mTBI (p = 0.11 using a two-sided T-test). For right button presses, reaction times were 0.54 ± 0.10 s for controls and 0.53 ± 0.07 for mTBI (p = 0.82 using a 2-sided T-test).)

### 2.4 Data analysis

Following collection and pre-processing, the data analysis pipeline was similar to that introduced by Seedat et al. (2020).

#### 2.4.1 Source localisation

We characterised brain activity at 78 cortical regions (Gong et al., 2009) defined according to the Automated Anatomical Labelling Atlas (AAL) (Tzourio-Mazoyer et al., 2002). To this end, we used a linearly constrained minimum variance (LCMV) beamformer (Van Veen et al., 1997) implemented in FieldTrip (Oostenveld et al., 2011), which estimates electrophysiological activity at a predefined location/orientation in the brain, whilst minimising contributions from all other sources (both in the brain and environmental interference). These estimates are known as virtual sensor time series. A single virtual sensor was placed at the centre of mass of each of the 78 AAL regions. A single shell head model (Nolte, 2003) was used to construct the forward solution for each participant by co-registering the MEG sensor locations onto an anatomical T1-weighted, age-appropriate MRI template (MNI152) using SPM12 through FieldTrip. A common spatial filter was computed for each region, using all artefact-free trials to generate a covariance matrix. The covariance matrix was regularised with 5% Tikhonov regularisation. The beamformer was applied to reconstruct the broadband time series for the centroid of each of the AAL regions. Sources were projected to the dominant orientation by taking the eigenvector of the source covariance with the largest eigenvalue (Sekihara et al., 2004). The reconstructed virtual time series were frequency filtered between 1 and 48 Hz. The resulting data comprise 78 regional electrophysiological time courses, per subject.

One significant challenge in MEG connectivity estimation is that the ill-posed nature of the MEG inverse problem causes “leakage” of signal between virtual sensors at separate locations (Brookes et al., 2012). This leakage manifests as a zero-time lag linear summation of activity from other regions and, for this reason, orthogonalisation of virtual sensors before connectivity calculation results in a marked reduction of leakage, albeit at the cost of true zero-phase-lag connectivity (O’Neill et al., 2015a). Here, we employed a multivariate symmetric orthogonalisation (Colclough et al., 2015) involving two steps: First, a set of orthonormal time-courses that are closest to the virtual sensor data, and for which there is a simple analytic solution, is found. Second, the solution is finessed by iteratively adjusting the lengths of the orthogonal vectors until the solution is as close as possible to the uncorrected time courses. The resulting data contain the 78 orthogonalised virtual sensor time series. Following application of this procedure, the time courses were downsampled to a 100 Hz sampling rate, mean centred, and variance normalised.

#### 2.4.2 Hidden Markov Model:

All 78 regional time courses for each subject were processed independently using a univariate time-delay embedded HMM. The details of the HMM have been described extensively in previous papers and will not be repeated here. Briefly, the HMM assumes that a series of mutually exclusive hidden “states” governs each electrophysiological time course. This means that for every brain region, each time point is associated with a single state. The sequence is Markovian (meaning that the state modelled is dependent only on the state immediately preceding it). In its simplest form, the HMM might describe each state by a Gaussian distribution, from which the MEG data are extracted. If at some time point *t*, the observed MEG data are most likely drawn from the Gaussian describing state 1, then state 1 would be assigned to time point *t*. Here, distinct from a simple Gaussian observation model, we used an HMM with time-delay embedding (Vidaurre et al., 2018) where each state is characterised by a different autocovariance pattern defined over a specified time window (of duration 230 ms). These state autocovariance patterns contain the spectral information of the signal when a particular state is active and consequently states are derived based upon specific repeating spectro-temporal patterns of activity. Previous work has shown that using the HMM in this way enables accurate identification of the pattern associated with the pan-spectral bursts that underlie the beta oscillatory signal (Seedat et al., 2020).

The HMM was applied as in Seedat et al. (Seedat et al., 2020). Specifically, model inference was undertaken using a variational Bayesian method. For each of the 78 time courses, we assumed 3 states, and so the model output was a set of 3 time courses representing the probability of each state being active over all time. To identify which of the three states corresponded to pan-spectral bursts (henceforth termed the “*burst state*”), we measured the correlation between the state probability and the amplitude of beta oscillations (defined by the application of a (Morlet-based) continuous wavelet transform to the regional time course and extracting those values corresponding to the 13–30 Hz frequency band). The state whose probability time course correlated highest with the beta envelope was taken as the burst state while the remaining 2 states were defined to be non-burst states. The probability time courses for each state were subsequently binarised by assuming that if the probability exceeded two thirds, then the given state had been entered. These binary time courses enabled the identification of bursts and post-hoc analysis allowed measurements such as burst (and non-burst) state duration, burst amplitude, and coincidence. This method was applied to each of the 78 time courses independently, for all subjects and tasks.

#### 2.4.3 Summary metrics for resting-state data:

For the resting-state data, we aimed to use the HMM output to investigate whether the previously observed (Zhang et al., 2020) beta deficits in power and connectivity could be explained in terms of our burst framework. We calculated the following features from the HMM output:

• **Burst amplitude:** The maximum value of the beta envelope during each visit to the burst state.
• **Total burst time:** The proportion of time spent in the burst state throughout the resting state recording. (Calculated as the total time burst state was active, divided by the total experimental duration.)
• **Burst duration:** The average time spent in the burst state, on each visit.
• **Burst count:** The number of visits to the burst state per second, calculated by counting the total number of visits in a recording and dividing by the total length of the recording.
• **Functional connectivity.** Here we define functional connectivity as the temporal coincidence between bursts in two regions. Burst coincidence between regions *i* and *j* was measured using the Jaccard index, $J^{i,j}$ – a ratio of the intersection over the union of two binary state time-courses. This yields a value between 0 and 1; 1 means perfect coincidence; 0 means no coincidence. Mathematically, $J^{i,j}=\sum_{t}^{N_{t}}{(B}_{t}^{i}\wedge B_{t}^{j})/\sum_{t}^{N_{t}}{(B}_{t}^{i}\vee B_{t}^{j})$ , where $B_{t}^{i}$ indicates whether the burst state was occupied in region i at time point t (1 indicating burst, 0 indicating non-burst). $N_{t}$ is the number of samples, and ∧ and ∨ are the logical AND and OR operators respectively.

#### 2.4.4 Machine learning analysis of burst coincidence connectomes

The burst coincidence connectomes derived from the resting state recordings were further analysed using a machine learning pipeline with the aim of determining their utility in distinguishing subjects with mTBI from healthy subjects. The pipeline included Recursive Random Forest Feature Selection (rRF-FS) and binary classification using a support vector machine (SVM) model. All possible, unique connections between the 78 regions of the AAL atlas ((78x77)/2 = 3003 connections) were used as input features. Using 10-fold cross-validation to evaluate classification performance, the rRF-FS was used to select the most important connections via a variable importance threshold, and consensus voting procedure (See (Zhang et al., 2020, Zhang et al., 2016) for more details), before SVM classification. Model performance was measured via the mean area under the receiver operating characteristic curve (ROC-AUC) after each cross-validation iteration. The statistical significance of the SVM model was tested by repeating the SVM training and classification 100 times, randomly permuting the sample group labels. Making use of the excellent interpretability of Random Forest Feature Selection, we assessed whether the chosen connections are among those driving the global reduction in burst connectivity.

#### 2.4.5 Summary metrics for task data:

For the task data, we used a similar approach, again measuring burst characteristics and connectivity, but this time in the context of task timing and the well-known beta band features (the movement-related beta decrease (MRBD) and the post-movement beta rebound (PMBR)). We calculated the following:

• **Burst probability time courses:** For a single region, binary burst time courses were reshaped into a matrix of time (within a trial) by the number of trials. For each trial timepoint, we assessed the probability (across trials) that a burst occurred. This was calculated as simply the sum of the total number of trials showing a burst at time t, divided by the total number of trials. These probability time courses were calculated within each subject and then averaged across subjects. We expected to see a decrease in burst probability during movement (corresponding to the MRBD) and an increase on movement cessation (delineating the PMBR).
• **Burst statistics during PMBR window**: The PMBR window was defined from 0.45 s to 0.85 s relative to the button press (Pfurtscheller et al., 1996). For each trial in every subject, burst amplitude and duration were calculated for each burst which fell within, began, or ended during the PMBR window. These values were averaged within each trial, and subsequently across trials and subjects.
• **Burst coincidence time courses:** The time evolution of the burst coincidence was generated by expanding on the Jaccard index method. For every pair of regions, the binary burst time courses for both regions were reshaped into a matrix of time within a trial, by the number of trials. The Jaccard index was then calculated for each time point, t, within a trial (i.e. rather than calculating the Jaccard index over all time as was done above in the functional connectivity section, we calculated it over all trials for each time point within a trial). This enabled us to define a time course of burst coincidence probability.

### 2.5 Statistical testing:

#### 2.5.1 Resting-state data

The resting-state HMM yielded 78 values of burst amplitude, non-burst amplitude, and total burst time. To avoid a-priori assumptions on the brain regions or connections implicated in mTBI (which may differ between subjects depending on the nature of the injury) we collapsed these metrics across all regions/connections. This left a single global mean value for each metric. We then computed the difference between groups (mTBI and controls) and assessed statistical significance using a non-parametric Wilcoxon rank-sum test, corrected for multiple comparisons across the three measures using the Benjamini-Hochberg procedure (Benjamini and Hochberg, 1995). This enabled direct testing of the hypothesis that *previous findings of abnormal beta activity can be explained by abnormalities in burst amplitude.*

Independently, our connectivity analyses generated 3003 values of connectivity (i.e. one value for each connection, between all possible pairs of the 78 AAL regions). Again, to avoid a-priori assumptions on the most prominent connections affected by mTBI, values were collapsed across all connections, and the global mean connectivity was calculated. This was done for all subjects and a Wilcoxon rank-sum test was used to test significance. This allowed us to test the hypothesis that *abnormalities in connectivity can be explained by a lack of coordination between bursts in spatially separate brain regions.*

#### 2.5.2 Motor task data

For the task paradigm, we first focused on the PMBR as this has been shown to be a sensitive marker of network communication and connectivity. We set out to test the hypothesis that interhemispheric connectivity would be disrupted in mTBI (i.e. by damage to white matter tracts in the corpus callosum). Specifically, we expected burst structure to be deficient during the PMBR, manifesting in changes of the burst features as well as synchrony between bursts.

We calculated the mean burst probability and burst amplitude during the PMBR. This was done separately for the left and right motor cortices, and left button press and right button press – yielding a total of 8 measurements. For completeness, we also measured the overall modulation of burst probability (i.e. the difference in burst probability between the PMBR and the MRBD windows). In all cases, a Wilcoxon rank-sum test was used to test for significant differences between groups (p < 0.05) and FDR correction was used to correct for multiple comparisons across the 12 separate tests (left/right cortex; left/right button press; 3 separate metrics). This allowed for testing of the hypothesis that *the bursts which drive the measurable PMBR will be altered in individuals with mTBI*.

To test our hypothesis that *connectivity between motor regions during the PMBR will be diminished in the patient cohort,* the group difference in the mean connectivity between the left and right motor cortices during the PMBR window was calculated, and statistical significance was assigned using a Wilcoxon rank-sum test. Here, an average was calculated over both trial conditions.

#### 2.5.3 Relationship with symptoms

Finally, the relationships between symptom severity – as measured via the SCAT2 – and MEG derived metrics (global burst amplitude, and burst connectivity) in the resting state, were assessed using Spearman correlation. We reasoned that burst amplitude and connectivity might have a monotonically decreasing relationship with symptom severity (i.e., those with more severe symptoms would have diminished burst amplitude and connectivity). To test this, *within the mTBI group only*, we calculated the correlation of symptom severity and MEG measures. P < 0.05 and FDR correction was used to determine statistical significance. The association between symptom severity and the mean connectivity of the connections selected via the rRF-FS procedure was also measured using Spearman correlation, to determine whether the subset of features would show a stronger relationship than the whole brain measure of connectivity. In addition, we also tested the same correlation across the *combined group* of subjects (i.e., patients and controls). Note that this combined measure doesn’t suggest a relationship between symptoms and MEG measures at an individual level. Rather, a significant correlation would be likely to be driven solely by a group difference. For this reason, the combined Spearman correlations were only used to support the group observations described above.

## 3 Results

### 3.1 Spontaneous beta bursts are abnormal in mTBI

**Fig. 2:**
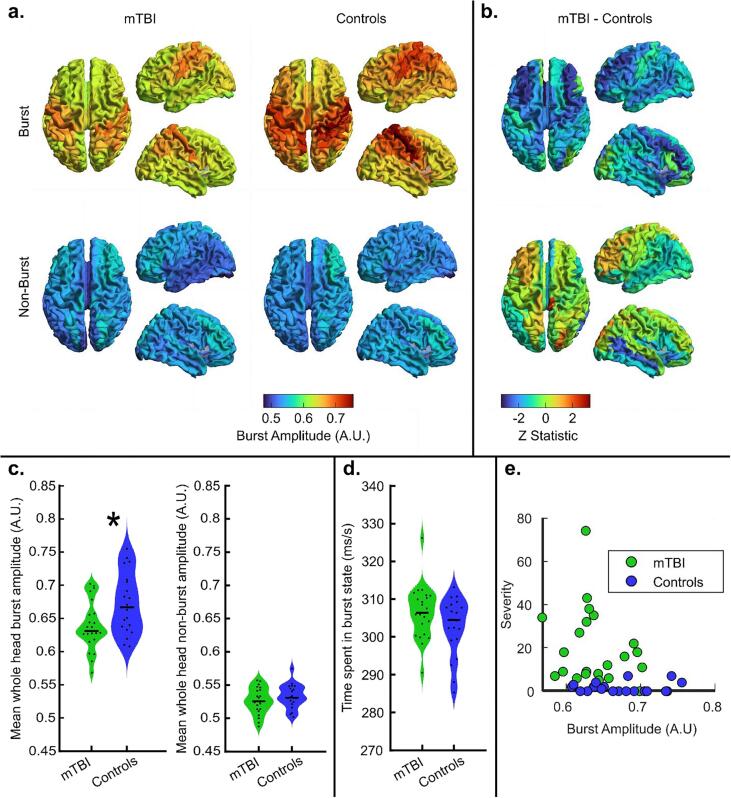
Resting-state burst statistics. a) The upper panel shows the spatial distribution of beta amplitude during bursts. The lower panel shows beta amplitude in the non-burst windows. In both cases, patients are shown on the left and controls on the right. b) Spatial signature of the differences in beta amplitude during bursts (top) and non-burst periods (bottom) (i.e. the difference between patients and controls, left and right in a) respectively) c) The left panel shows average burst amplitude, the right panel shows beta amplitude in the non-burst states, demonstrating no significant difference between patients and controls. In both cases, results are collapsed across 78 brain regions and each data point represents an individual subject. Note that burst beta amplitude is significantly (p = 0.016; Wilcoxon sum rank test) diminished in patients. d) Violin plot showing the average time spent in the burst state (no significant difference between groups) e) Scatter plot of global burst amplitude versus SCAT-2 symptom severity. Spearman correlation for the combined data points (patients and controls) gave R = -0.399; p = 0.0088. For the patients only, we found R = -0.189; p = 0.387.

Fig. 2 shows the burst statistics captured during the resting state recording. In Fig. 2**a**, the upper panel shows the spatial distribution of beta amplitude during the bursts identified by the HMM. In agreement with previous work (Seedat et al., 2020), in control subjects the amplitude is maximal over the posterior frontal and parietal lobe, in particular the sensorimotor cortices. This amplitude appears diminished in the mTBI group although the spatial pattern is similar. The lower panel of Fig. 2a shows beta amplitude during the non-burst states. Here, the overall amplitude is much lower (as expected) and the spatial pattern is no longer apparent. Fig. 2b shows the corresponding spatial distributions of the differences in the burst (upper plot) and non-burst (lower plot) beta amplitude between the two groups.

In Fig. 2c, the violin plots show the distribution (across subjects) of whole-brain burst and non-burst amplitudes; the plot on the left shows burst amplitude; the plot on the right shows the amplitude during the non-burst periods. Note that, in agreement with our hypothesis, during bursts, there is a significant (p = 0.0165; Wilcoxon sum rank test) drop in amplitude for patients relative to controls. However, here there is no such measurable difference in the non-burst windows. This demonstrates an important point: in (Zhang et al., 2020) the authors showed diminished resting-state beta amplitude; here we extend this finding to show that this reduction is driven largely by the spectral content of the bursts, and specifically that the amplitude of the beta component is reduced. Results have been collapsed across all brain regions and each of the black data points represents a different subject. Burst amplitude was also significantly correlated with symptom severity when measured across the whole cohort (combining patients and controls) (Spearman R = − 0.399, p = 0.0088) (Fig. 2e. Fig. 2d contains violin plots showing the average time spent in the burst state. Note that there is no significant difference between patients and controls; further suggesting that the beta deficit in patients stems from a reduction in burst amplitude and not a reduction in the number or density of occurrence of the bursts. Finally, Fig. 2e shows a scatter plot of global burst amplitude versus SCAT-2 symptom severity. Spearman correlation for the combined data points (patients and controls) gave R = -0.399; p = 0.0088, which is likely driven by the difference between groups. However, for the patients only, we found no evidence (R = -0.189; p = 0.387, Spearman Correlation) of a direct correlation between global burst amplitude and symptom severity. Note that we also tested the correlation between our burst metric and a more traditional approach to measuring beta amplitude. In the latter, a Morlet-wavelet transform was applied to the beamformer reconstructed data and values corresponding to the canonical beta band (13–30 Hz) were extracted to obtain the amplitude envelope. The mean envelope value was then computed (over the entire experimental duration) and averaged across brain regions. We found a significant correlation (R = 0.93; p = 5x10-20; Pearson correlation) between burst amplitude and beta amplitude (over all time), demonstrating clearly that the traditional beta metric (collapsed over all time) is at least partly driven by the beta amplitude during the burst windows (which only account for ∼ 30% of the overall experimental duration).

**Fig. 3:**
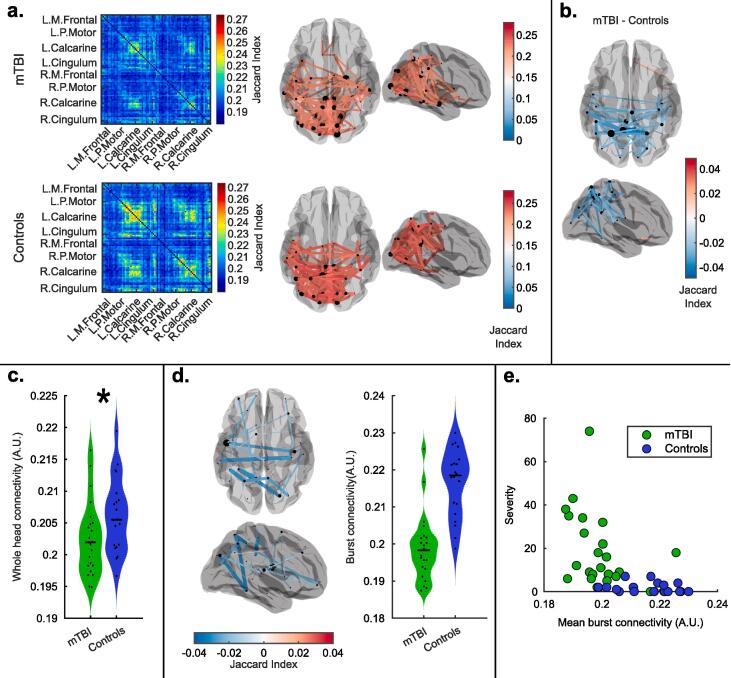
Resting-state functional connectivity. a) Resting-state connectome matrices for patients (upper panel) and controls (lower panel). In both cases, connectivity is calculated via the assessment of burst coincidence between regions. The glass brains show the spatial structure of the information in the corresponding connectome matrices. The red lines show 5% of connections with the highest functional connectivity value. b) The glass brain plot shows the 2% of connections with the largest difference between patients and controls. c) Whole-brain functional connectivity assessment in patients and controls. Each data point represents the average connectivity across all connections, for a single individual (i.e. the overall sum of all of the matrix elements in the connectomes shown in (a) divided by the number of connections). Whole head connectivity – computed via assessment of burst coincidence – is significantly (p = 0.031; Wilcoxon sum rank test) diminished in patients. d) Glass brain showing the connections selected using recursive Random Forest Feature selection and violin plot showing the average connectivity for those connections in both groups. e) Scatter plot showing the relationship between symptom severity and burst connectivity in the rRF-FS selected connections. Spearman correlation for the combined data points gave R = -0.72; p = 8x10^-8^. Spearman correlation for the mTBI group only gave R = -0.45; p = 0.03. (For interpretation of the references to colour in this figure legend, the reader is referred to the web version of this article.)

Fig. 3 shows the results of functional connectivity assessment in resting-state data. Fig. 3a shows connectome matrices calculated via assessment of burst coincidence in patients (upper left panel) and controls (lower left panel). The corresponding upper and lower right panels show the spatial distribution of the 5% of connections (i.e. the 3003*0.05 ∼ 150 connections) with the highest Jaccard index, plotted on a glass brain. The result for controls mirrors previous findings (both calculated using burst coincidence (Seedat et al., 2020) and the more widely used amplitude correlation metrics (Hunt et al., 2016)); the largest connectivity tends to be found between homologous regions of the occipital, motor, sensory, and posterior parietal cortices. Interestingly, the overall connectivity pattern was maintained in patients, but the absolute values of connectivity are diminished.

Fig. 3b shows the spatial signature of the differences in connectivity between patients and controls. Here we plot the 2% of connections with the highest differences between groups. The spatial signature shows that the largest resting-state connectivity differences are between posterior parietal regions. Note again that these findings extend previous work; Zhang et al. demonstrated using ‘classical’ methods that beta connectivity is diminished in mTBI. Here, using the same data, we show that these differences can be explained by assessment of burst coincidence between brain regions.

This effect is formalised in the violin plot in Fig. 3c. Here, each data point represents the mean strength of all connections in a single subject, and we note that connectivity is diminished significantly (p = 0.031; Wilcoxon rank sum test) in the mTBI group relative to controls. A significant correlation between symptom severity and global burst connectivity was also found (correlation derived using data from the mTBI group and controls) further verifying this measure (Spearman R = -0.39; p = 0.01). However, when taking into account individuals with mTBI only, the correlation with symptomology was not significant (Spearman R = -0.327; p = 0.128). See the supplementary materials for a scatter plot of global burst connectivity against severity.

The glass brain plot in Fig. 3d shows the connections which most accurately separate patients and controls, as selected using the data-driven rRF-FS approach. The violin plot shows the mean connectivity over the selected features (connections) for each subject. Note that the separation between the groups is greatly improved compared to the global measure, as would be expected from this type of ML approach. Interestingly, many of the connections which distinguish patients are interhemispheric, potentially implicating damage in the corpus callosum. The average ROC-AUC classification accuracy across the ten cross-validation folds was 0.98 with a SD of 0.08. Finally, we measured a significant correlation between symptom severity and the average burst coincidence of the rRF-FS features; for the combined mTBI and control groups we found (R = -0.72; p = 8x10^-8^; Spearman Correlation). However, we note that this is influenced by the ML approach which yields features that best differentiate the groups and might therefore inflate the value of the calculated Spearman correlation. More interestingly, we found that for the mTBI group only, there was a significant correlation between connectivity and symptom severity (R = -0.45; p = 0.03; Spearman Correlation) which could not have been driven by the ML approach. A scatter plot of symptom severity against rRF-FS selected burst connectivity is shown in Fig. 3e.

### 3.2 MTBI disrupts the dynamic neural repertoire of the motor system

**Fig. 4:**
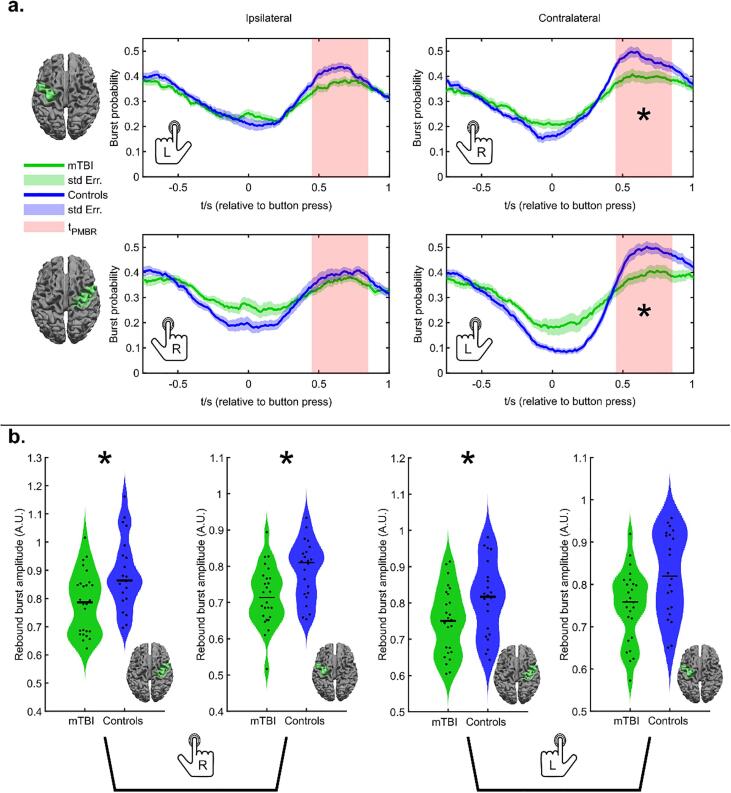
Burst statistics during a motor task. a) Time courses showing the probability of bursts throughout a button press. The upper panel shows the case for the left primary motor cortex during a left hand (left) and right hand (right) movement. The lower panel shows the case for the right primary motor cortex during a right hand (left) and left hand (right) movement. b) Burst amplitude in the beta band during the post-movement beta rebound. Left-hand plots show contralateral and ipsilateral cortices during a left-hand button press. Right-hand plots show ipsilateral and contralateral cortices during a right-hand button press. * indicates statistical significance (p < 0.05*; Wilcoxon Rank Sum test) following FDR correction for multiple comparisons.

Fig. 4 shows burst statistics during the motor task. Note that the task contained both left and right finger movements and these have been analysed separately. We also examine effects in both contra- and ipsilateral cortices. (Statistical analyses employed FDR correction to account for multiple comparisons). Fig. 4a shows the temporal evolution of burst probability throughout the task. The upper two plots show burst probability in the left motor cortex, for left (left) and right (right) button presses. The lower two plots show burst probability in the right motor cortex, for right (left) and left (right) button presses. In all cases, the green trace shows the patients and the blue trace shows healthy controls. The solid lines show the mean across subjects and the shaded regions show the standard error across subjects. Notice that a characteristic response is seen in both regions and all conditions, whereby the burst probability is diminished during the movement itself (i.e. around time t = 0) and is enhanced immediately following the movement. Previous work (Little et al., 2019) has shown that this change represents the basis of the MRBD and PMBR. There does appear to be a systematic effect in the contralateral cortex whereby the modulation of the burst probability is lower in patients.

In testing the burst probability during the rebound window, we found a significantly diminished likelihood of bursts in contralateral cortices (Right motor, left button p = 0.0099*; Left motor, right button p = 0.0248*; Wilcoxon Rank Sum test) but not ipsilateral cortex (* indicates significance following FDR correction). The overall modulation of burst probability – as measured by the difference in probability in the rebound and desynchronization time periods – was also significantly different in both conditions in the contralateral cortex; (left motor cortex, right press: p = 0.024*; right motor cortex, left press: p = 0.0047*; Wilcoxon Rank Sum test) In ipsilateral cortex modulation of burst probability was not significant following multiple comparison correction.

Fig. 4b shows the amplitude during the HMM identified bursts during the rebound window. Here, we find significantly reduced beta burst amplitude in both left and right motor cortex during left button presses: (left-hand movement; left motor cortex, p = 0.007*. Left-hand movement; right motor cortex, p = 0.005*; Wilcoxon Rank Sum test). Similar trends were also observed for right-hand button presses but this was only significant in the right motor cortex (p = 0.0003*; Wilcoxon Rank Sum test).

Overall, these results support our previous findings shown in Fig. 2, demonstrating significantly reduced burst amplitude in the beta frequency band. Correlation analysis of symptom severity with burst amplitude in the contralateral and ipsilateral motor cortices found no significant relationship after correcting for multiple comparisons.

**Fig. 5:**
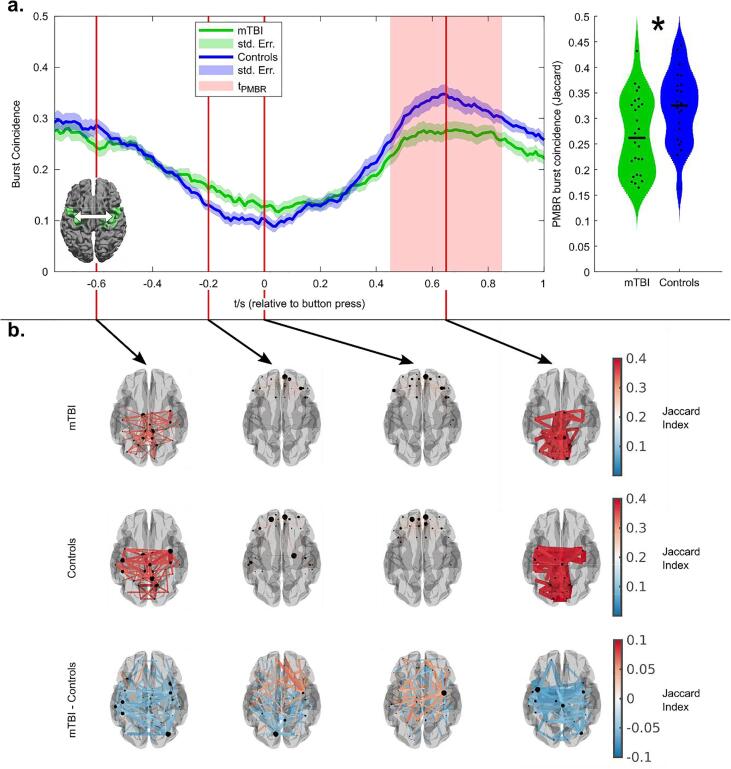
Functional connectivity during a motor task. a) Time course showing the temporal evolution of burst coincidence throughout finger movement. Note that burst coincidence is significantly (p = 0.021; Wilcoxon rank sum test) less likely during the beta rebound window, in patients relative to controls. b) The spatial distribution of dominant connections at 4 time points during the task. The upper set shows the case for patients. The centre set shows the case for controls, and the lower set shows the dominant differences. In all cases, the 2% of connections with the highest values are shown. Notice that during the rebound window, the dominant differences are between bilateral motor regions. See the supplementary materials for a video showing the whole time evolution of the functional connections.

Fig. 5 shows transient functional network patterns during finger movement. Fig. 5a shows the temporal evolution of burst coincidence between the left and right primary motor cortex, during the task. The green trace shows the patients and the blue trace shows healthy controls. In agreement with previous work (O’Neill et al., 2015b), we observe lower connectivity during unilateral movement, followed by an increase on movement cessation; this supports the theory that the PMBR is a time of elevated connectivity between primary cortices and other regions (Tewarie et al., 2019), and carries a top-down inhibitory influence. The largest difference between patients and controls, in terms of connectivity, occurs during the post-movement rebound period. To test this statistically, the violin plot on the right-hand side shows connectivity estimated during the rebound window, demonstrating a significant (p = 0.021; Wilcoxon rank sum test) reduction.

Finally, Fig. 5b shows the spatial signature of the dominant (2%) of functional connections at 4 selected time points during the task. It is interesting to note how this spatial pattern changes considerably during the task with posterior parietal and sensorimotor connections, apparent at the start, giving way to frontal connectivity, which then, in turn, gives way to dominant interhemispheric motor network connectivity during the rebound window. The upper set of images in Fig. 5b show the case for patients, the middle set for controls, and the lower set show the highest (2%) of differences between the two groups. Note that these differences map out a clear motor network around the time of the post-movement rebound, showing a clear and significant reduction in burst coordination within this time window. No significant correlation between symptom severity and PMBR connectivity (between the motor cortices) was found (combined groups: R = -0.296; p = 0.043, patients only: R = -0.005; p = 0.98; Spearman Correlation. See supplementary information for a scatter plot).

## 4 Discussion

MTBI is now recognised as a ‘silent epidemic’ and a serious public health concern. A minority, but substantial number, given the high incidence of mTBI, suffer from persistent post-concussive symptoms and can experience chronic, lifelong deficits that result in reduced quality of life. The insidious long term health effects of mTBI are of great concern in the world of contact sports, such as rugby and American Football, where increasing evidence suggests repeated mTBI and sub-concussive blows can precipitate cognitive decline and even neurodegenerative disease, such as early-onset dementia. (Costanza et al., 2011, Hume et al., 2017, Pearce et al., 2018)

### 4.1 A summary of findings:

Our resting-state findings demonstrated abnormalities in both beta burst amplitude and the coincident bursting that mediates connectivity. In Fig. 2, results show clearly that the diminishment of beta amplitude in patients is not general over all time; rather it is mostly a property of bursts. Indeed, when the burst state was active, significant differences between patients and controls could be found which were not mirrored during non-burst windows; this means that lower beta amplitude is occurring, approximately one-third of the time. It is important to note that the null result in the non-burst windows does not necessarily imply that there is no information of interest outside the bursts; indeed, a more subtle analysis, perhaps looking at individual brain regions, may well demonstrate significant differences between patients and controls. Nevertheless, at least at the whole-brain level, our results suggest that the largest difference in beta amplitude between patients and controls occurs during the active burst state. Again, using resting-state data, we were able to show that the previous findings of reduced connectivity (Zhang et al., 2020) – measurements made using amplitude envelope correlation – are driven by a drop in burst coordination across the brain (see Fig. 3. Whilst we showed that connectivity over the whole brain was diminished in mTBI, post hoc analyses suggested that the most affected connections were interhemispheric, between regions of polymodal parietal cortices. These regions are well-known foci of sensorimotor integration and contain dense connections with numerous brain areas (Whitlock, 2017). We saw a significant correlation between connectivity and symptom severity in patients, following the selection of a subset of connections using the rRF approach. This demonstrates the utility of this data-driven method to determine which connections are most affected. This result should be used to inform the hypotheses of future studies, potentially leading to stronger results.

In our task data, we probed more specific hypotheses relating to the MRBD and PMBR. These effects have been observed in electrophysiological imaging data for many years and are one of the most robust findings in neuroscience. Recent work (Tewarie et al., 2019) has linked the PMBR to enhanced connectivity within a wider network encompassing primary motor and motor planning regions, with the implication that this network provides top-down inhibitory influence to “shut down” and recalibrate the motor cortex post-movement. Given the global and inter-hemispheric nature of this network, we hypothesised that white matter damage might make the PMBR abnormal in mTBI patients. Results supported these hypotheses, showing diminished modulation of burst probability, lower amplitude, and a deficit in coincident bursts, during the PMBR in patients. Whilst it remains a possibility that this effect was due to the way in which the task was performed (e.g., it’s conceivable that patients and controls may move differently), there was no measurable difference in reaction times between groups and so it’s likely that the observed deficit is a fundamental feature of mTBI. Interestingly these motor-specific findings did not correlate significantly with overall symptom severity, however, this may be because our symptom scores are global, and we did not take correlate them with an objective assessment of motor function specifically. Previously, mTBI sequelae have been demonstrated to include motor deficit (Pearce et al., 2018), and these findings represent a putative mechanism.

### 4.2 Mechanistic interpretations: The importance of beta band phenomena

Despite the unmet clinical need for rapid and reliable biomarkers for mTBI, imaging signatures for concussive injuries are difficult to establish given the heterogeneity of mechanism, injury, symptoms, and long-term outcome. Neurophysiological indices derived from MEG have shown significant promise in their sensitivity and specificity in the assessment of mTBI. Previous work (Huang et al., 2014) has demonstrated that abnormal neural oscillations, particularly in the low-frequency delta band, can be used to successfully distinguish mTBI patients from healthy controls. More recent works (Huang et al., 2020, Zhang et al., 2020) show that higher frequency oscillations (beta and gamma) are reliable markers too, as well as emerging measures of electrophysiological dynamics including functional connectivity. However, these previous measures have assumed the ‘classical’ picture that oscillations apparent in time-averaged brain signals represent ongoing rhythmic processes. In this paper, for the first time, we used the emerging theory of pan-spectral bursts – temporally discrete, transient brain dynamics – elucidated using a Hidden Markov Model, to further this field and show the multiscale, multiterminal impact of a single mTBI on dynamic neural activity. Our results, which show intrinsic and spontaneous bursting dynamics in the beta range are diminished, suggest that the neuronal assemblies responsible for generating transient beta events are affected by mTBI. This finding mirrors, in part, recent findings in fMRI which report altered dynamics and signal complexity after mTBI (Churchill et al., 2020). A decrease in burst amplitude might be caused by a reduction in excitatory drive from the thalamus (Sherman et al., 2016) which would support the notion that long-range connections to the cortex are affected by concussive events. Our finding that diminished beta band connectivity in mTBI is due to a lack of burst coordination in long-range networks (at rest and during a task), particularly within inter-hemispheric connections in the parietal lobes, could be explained by a variety of mechanisms. These could include axonal damage with possible subsequent Wallerian degeneration, reduced myelination and other forms of neurodegeneration affecting neural synchrony.

Changes in beta band power and disruption of beta band connectivity at rest, as well as beta abnormalities during motor tasks, have been reported in patients with neurodegenerative disease such as Parkinson’s disease (Bosboom et al., 2006, Heinrichs-Graham et al., 2014, McColgan et al., 2020, Stoffers et al., 2007). Recent reports on a cohort of subjects with repeated sports-related mTBI provide evidence of increased inflammation and accumulation of Tau protein (which is thought to contribute to the development of neurodegenerative diseases) (Marklund et al., 2021). Given the likely connection between mTBI and chronic neurodegenerative disease (Gardner and Yaffe, 2015) the beta band is of particular relevance to mTBI. This relevance is further implied by studies (e.g.(Brookes et al., 2011, Hipp et al., 2012)) demonstrating that canonical brain networks – including those associated with sensory/motor function, and the higher order attentional networks – can be elucidated via an analysis of beta-band oscillations. These findings are in broad agreement with other emerging theories that beta oscillations carry top-down inhibitory influence on primary cortices. They also agree with a predictive coding model which suggests that beta oscillations carry internal forward models (whilst gamma oscillations reflect prediction errors) (see e.g. (Bastos et al., 2015, Buschman and Miller, 2007, Kopell et al., 2000)). Taken together, we propose that beta band processes support, and offer a sensitive measure of, long-range communication and connectivity. In mTBI, it is hypothesised that the diffuse axonal injury (DAI) (Gazdzinski et al., 2020) and in particular disruption of the white matter around the corpus callosum, is one of the main drivers of neuropathology. This being the case it is likely that the long-range networks (particularly interhemispheric) mediated by beta oscillations would be disrupted by injury. It is therefore likely that assessment of beta band phenomena would provide a marker of mTBI. Indeed, such a marker would reflect the disruption of the connectome (either localised coup- and countercoup injury or diffuse injury) which is likely to exist in mTBI (Browne et al., 2011, Kirov et al., 2013) but is too subtle to be measured reliably using clinically available structural imaging. Additionally, mTBI can cause a plethora of neurochemical changes, impact neurotransmission and cause long-lasting metabolic impairment (see (MacFarlane and Glenn, 2015) for a review), which could disturb the effective generation of burst activity. Again, this is measurable using functional imaging but will escape detection in structural imaging. An important consideration here is time post-injury; neurochemical and neurometabolic changes, and their recovery to normal levels, vary in time scales, with some hyperacute effects resolving within minutes (e.g. K^+^ ion efflux and altered glutamate levels) and others remaining altered for several days (e.g. Ca^2+^ accumulation) (Churchill et al., 2020, Giza and Hovda, 2014, Jamjoom et al., 2021, MacFarlane and Glenn, 2015). In the long term, inflammation and glial activation in the subcortical white matter but also changes in myelination, Wallerian degeneration, or oedema, might disrupt functional connectivity. Longitudinal, multimodal studies covering time points soon after injury are required to produce a clear picture of how electrophysiological signals are altered during recovery.

Whilst previous studies in this area (Huang et al., 2020, Zhang et al., 2020) employ the classical measure of oscillatory amplitude, here we chose to employ the “burst” framework. The burst model of beta has gained traction in the neuroscientific community in recent years, not only as a conceptual model but moreover because it has spawned some of the first mathematical models suggesting how beta oscillations might be generated physiologically (Sherman et al., 2016). This, therefore, affords the possibility that, in time, we can link the putative beta band markers of mTBI to a neurophysiological model. One consequence of the bursting hypothesis is that beta effects, whilst typically thought to be ongoing over all time, only occur for a small percentage of the overall duration of a MEG scan. It is with this in mind that we might expect a bursting framework to offer a more sensitive measure than classical analyses, where the effects sought might become averaged out over time.

### 4.3 Clinical perspective

Previous papers have tended to focus on differentiation between individuals with mTBI and controls, with recent findings offering between 80 and 100% classification accuracy using ML. In the current work, we have also employed an ML approach – applied only to the connectivity data – which shows a reasonably high classification accuracy. This shows that the putative biomarkers of mTBI outlined here might also offer diagnostic capability. However, mTBI remains a diagnosis based on clinical history, patient experience, self-reported symptoms, and is ultimately delivered by medically qualified clinicians. A brain scan, however accurate, that adds nothing other than to confirm the diagnosis, is of little practical help when determining a treatment pathway. Instead, we would argue that significantly more import lies in 1) the ability to understand the sequelae of mTBI using hypothesis-driven objective assessment and 2) the ability of an imaging modality to be able to predict patient outcome or rehabilitation needs. MEG holds some promise in this area, but to realise its potential, we must employ the most sensitive markers of illness in a multivariate framework. While it is difficult to prevent overfitting of classifiers trained on relatively small datasets, employing feature selection techniques, such as the random forest approach outlined in this study, will be crucial in selecting candidate markers and will also aid hypothesis generation for future research. The burst deficits shown here could likely be combined with classical features and low-frequency observations into a single multi-variate biomarker. This may have predictive power to offer clinicians new tools in the management of this debilitating problem.

### 4.4 Limitations

There are several limitations to the current study which should be expounded. In terms of methodology, source localisation is impacted by the ill-posed nature of the MEG inverse problem. Brain regions, particularly those in close proximity, can exhibit signal leakage which will artificially inflate connectivity measures. To mitigate this, we applied orthogonalisation which eliminates zero phase lag effects; this is known to reduce artefactual connectivity but at the expense of real zero-phase-lag interactions (Brookes et al., 2012). This means that, whilst the long-range connections that we observe to differ between groups are real, there may be other (particularly short-range) connections that have been missed in this study due to this methodological limitation. A second technical limitation relates to the identification of the beta burst state. The method uses a univariate HMM in which three states are identified for each brain region. Following this, the state whose temporal occurrence most strongly correlates with high beta power is designated the burst state. This means that, hypothetically, if a brain region was dominated by noise, a burst state would still be selected (i.e., as that most correlated with the beta envelope, even if that correlation was low) and could be meaningless. Related, two regions exhibiting a coincident burst may not necessarily be exhibiting a functional connection. This is a fundamental limitation of the univariate nature of the method; however, we note that this same methodology has been applied successfully in previous papers (Gascoyne et al., 2021, Seedat et al., 2020); it is known to reliably extract connectome metrics (based on burst coincidence), and coincident bursts have been demonstrated to occur during periods of increased phase locking. For these reasons, it remains likely that the method offers a true picture of oscillatory dynamics and connectomics.

Additionally, two experimental limitations should be mentioned. Firstly, our motor task employed relatively short inter-stimulus intervals. The beta rebound is known to last for more than 7 s, and so the rebound following a trial will not be given a chance to fully relax before the following trial begins (Fry et al., 2016, Pakenham et al., 2020). We note that to gain a sufficient signal to noise ratio to characterise these effects, a large trial count is desirable; however, maintaining a large trial count whilst also allowing the rebound to fully relax between trials would lead to an experiment that was too long for patients to tolerate. Consequently, the experimental design used, whilst cofounded, is practical. A further limitation was the sample size used in our ML analysis. Using large numbers of features and a small sample size, it is conceivable that overfitting might occur when applying ML techniques such as the ones we have presented here. However, high classification accuracy does not imply overfitting in every case. As demonstrated in previous work which used a similar ML pipeline and sample size (Zhang et al., 2017, Zhang et al., 2016), the combination of feature reduction and 10-fold cross-validation allowed us to train SVMs which correctly classified the testing samples. Importantly, there was no information leakage between training and testing samples during 10-fold cross-validation as feature selection and reduction was conducted without including the testing samples during each of the folds. We are confident that the high classification accuracy is representative of what could be achieved in a larger study. However, we acknowledge that the relatively small sample size studied here remains a limitation and further samples are needed to ultimately validate this, and indeed all mTBI MEG findings. Finally, our study was limited by the behavioural measures that were acquired. Specifically, the SCAT2 is a generic questionnaire-based assessment that covers many areas. It remains a useful measure of symptomology but is not specific. This is likely why within-group correlations between MEG derived metrics were weak (in the case of connectivity), or absent (in all other cases). In the task, it is very unlikely that such a broad and general symptom score would be likely to correlate with the subtle MEG metrics that only relate to a single brain network. In future studies, behaviour should be more accurately assessed, potentially using motor specific tasks/questionnaires to better assess the relationships between neural measures and symptoms.

## 5 Conclusion

Here, for the first time, we show that fundamental and mechanistic neural bursting phenomena in the brain are disrupted by a single mTBI in the sub-acute phase following injury when structural MRI is normal. This dysregulated neural repertoire exists intrinsically and during dynamic recalibration of the motor system after behavioural output. These results point towards a mechanism whereby white matter damage disrupts network function; whilst the damage itself may be too subtle for structural imaging to see, its functional consequences are accessible using MEG.

### CRediT authorship contribution statement

**Lukas Rier:**Methodology, Software, Formal analysis, Writing – original draft,**Rouzbeh Zamyadi:**Investigation, Writing – review & editing,**Jing Zhang:**Software, Writing – review & editing,**Zahra Emami:**Investigation,**Zelekha A. Seedat:**Software, Writing – review & editing,**Sergiu Mocanu:**Software,**Lauren E. Gascoyne:**Writing – review & editing,**Christopher M. Allen:**Writing – review & editing,**John W. Scadding:**Software, Writing – review & editing,**Paul L. Furlong:**Writing – review & editing,**Gerard Gooding-Williams:**Writing – review & editing,**Mark W. Woolrich:**Writing – review & editing,**Nikos Evangelou:**Writing – review & editing,**Matthew J. Brookes:**Supervision, Conceptualization, Funding acquisition, Writing – original draft,**Benjamin T. Dunkley:**Supervision, Conceptualization, Funding acquisition, Writing – review & editing.

## References

[b0005] Aoki Y., Inokuchi R., Gunshin M., Yahagi N., Suwa H. (2012). Diffusion tensor imaging studies of mild traumatic brain injury: A meta-analysis. J. Neurol. Neurosurg. Psychiatry.

[b0010] Bastos A.M., Vezoli J., Bosman C.A., Schoffelen J.M., Oostenveld R., Dowdall J.R., DeWeerd P., Kennedy H., Fries P. (2015). Visual areas exert feedforward and feedback influences through distinct frequency channels. Neuron.

[b0015] Benjamini Y., Hochberg Y. (1995). Controlling the False Discovery Rate: A Practical and Powerful Approach to Multiple Testing. J. R. Stat. Soc. Ser. B.

[b0020] Bosboom J.L.W., Stoffers D., Stam C.J., van Dijk B.W., Verbunt J., Berendse H.W., Wolters E.C. (2006). Resting state oscillatory brain dynamics in Parkinson’s disease: An MEG study. Clin. Neurophysiol..

[b0025] Brookes M.J., Hale J.R., Zumer J.M., Stevenson C.M., Francis S.T., Barnes G.R., Owen J.P., Morris P.G., Nagarajan S.S. (2011). Measuring functional connectivity using MEG: Methodology and comparison with fcMRI. Neuroimage.

[b0030] Brookes M.J., Woolrich M.W., Barnes G.R. (2012). Measuring functional connectivity in MEG: A multivariate approach insensitive to linear source leakage. Neuroimage.

[b0035] Browne K.D., Chen X.H., Meaney D.F., Smith D.H. (2011). Mild traumatic brain injury and diffuse axonal injury in swine. J. Neurotrauma.

[b0040] Buschman T.J., Miller E.K. (2007). Top-down versus bottom-up control of attention in the prefrontal and posterior parietal cortices. Science (80-..

[b0045] Carroll L.J., Cassidy J.D., Holm L., Kraus J., Coronado V.G. (2004). Methodological issues and research recommendations for mild traumatic brain injury: The WHO Collaborating Centre Task Force on Mild Traumatic Brain Injury. J. Rehabil. Med. Suppl..

[b0050] Cassidy J.D., Carroll L.J., Peloso P.M., Borg J., von Holst H., Holm L., Kraus J., Coronado V.G. (2004). Incidence, risk factors and prevention of mild traumatic brain injury: Results of the WHO Collaborating Centre Task Force on Mild Traumatic Brain Injury. J. Rehabil. Med. Suppl..

[b0055] Churchill N.W., Hutchison M.G., Graham S.J., Schweizer T.A. (2020). Scale-free functional brain dynamics during recovery from sport-related concussion. Hum. Brain Mapp..

[b0060] Colclough G.L., Brookes M.J., Smith S.M., Woolrich M.W. (2015). A symmetric multivariate leakage correction for MEG connectomes. Neuroimage.

[b0065] Costanza A., Weber K., Gandy S., Bouras C., Hof P.R., Giannakopoulos P., Canuto A. (2011). Review: Contact sport-related chronic traumatic encephalopathy in the elderly: clinical expression and structural substrates. Neuropathol. Appl. Neurobiol..

[b0070] Fry A., Mullinger K.J., O’Neill G.C., Barratt E.L., Morris P.G., Bauer M., Folland J.P., Brookes M.J. (2016). Modulation of post-movement beta rebound by contraction force and rate of force development. Hum. Brain Mapp..

[b0075] Gardner R.C., Yaffe K. (2015). Epidemiology of mild traumatic brain injury and neurodegenerative disease. Mol. Cell. Neurosci..

[b0080] Gascoyne L.E., Brookes M.J., Rathnaiah M., Katshu M.Z.U.H., Koelewijn L., Williams G., Kumar J., Walters J.T.R., Seedat Z.A., Palaniyappan L., Deakin J.F.W., Singh K.D., Liddle P.F., Morris P.G. (2021). Motor-related oscillatory activity in schizophrenia according to phase of illness and clinical symptom severity. NeuroImage Clin..

[b0085] Gazdzinski L.M., Mellerup M., Wang T., Adel S.A.A., Lerch J.P., Sled J.G., Nieman B.J., Wheeler A.L. (2020). White Matter Changes Caused by Mild Traumatic Brain Injury in Mice Evaluated Using Neurite Orientation Dispersion and Density Imaging. J. Neurotrauma.

[b0090] Giza C.C., Hovda D.A. (2014). The New Neurometabolic Cascade of Concussion. Neurosurgery.

[b0095] Gloor, P., Ball, G., Schaul, N., 1977. Brain lesions that produce delta waves in the EEG. Neurology 27, 326–326. 10.1212/wnl.27.4.326. 10.1212/wnl.27.4.326 557774

[b0100] Gong G., Rosa-Neto P., Carbonell F., Chen Z.J., He Y., Evans A.C. (2009). Age- and gender-related differences in the cortical anatomical network. J. Neurosci..

[b0105] Heinrichs-Graham E., Wilson T.W., Santamaria P.M., Heithoff S.K., Torres-Russotto D., Hutter-Saunders J.A.L., Estes K.A., Meza J.L., Mosley R.L., Gendelman H.E. (2014). Neuromagnetic evidence of abnormal movement-related beta desynchronization in Parkinson’s disease. Cereb. Cortex.

[b0110] Hipp J.F., Hawellek D.J., Corbetta M., Siegel M., Engel A.K. (2012). Large-scale cortical correlation structure of spontaneous oscillatory activity. Nat. Neurosci..

[b0115] Huang M.-X., Huang C.W., Harrington D.L., Nichols S., Robb-Swan A., Angeles-Quinto A., Le L., Rimmele C., Drake A., Song T., Huang J.W., Clifford R., Ji Z., Cheng C.-K., Lerman I., Yurgil K.A., Lee R.R., Baker D.G. (2020). Marked Increases in Resting-State MEG Gamma-Band Activity in Combat-Related Mild Traumatic Brain Injury. Cereb. Cortex.

[b0120] Huang M.X., Nichols S., Baker D.G., Robb A., Angeles A., Yurgil K.A., Drake A., Levy M., Song T., McLay R., Theilmann R.J., Diwakar M., Risbrough V.B., Ji Z., Huang C.W., Chang D.G., Harrington D.L., Muzzatti L., Canive J.M., Christopher Edgar J., Chen Y.H., Lee R.R. (2014). Single-subject-based whole-brain MEG slow-wave imaging approach for detecting abnormality in patients with mild traumatic brain injury. NeuroImage Clin..

[b0125] Hume P.A., Theadom A., Lewis G.N., Quarrie K.L., Brown S.R., Hill R., Marshall S.W. (2017). A Comparison of Cognitive Function in Former Rugby Union Players Compared with Former Non-Contact-Sport Players and the Impact of Concussion History. Sport. Med..

[b0130] Hunt B.A.E.E., Tewarie P.K., Mougin O.E., Geades N., Jones D.K., Singh K.D., Morris P.G., Gowland P.A., Brookes M.J. (2016). Relationships between cortical myeloarchitecture and electrophysiological networks. Proc. Natl. Acad. Sci. U. S. A..

[b0135] James S.L., Bannick M.S., Montjoy-Venning W.C., Lucchesi L.R., Dandona L., Dandona R., Hawley C., Hay S.I., Jakovljevic M., Khalil I., Krohn K.J., Mokdad A.H., Naghavi M., Nichols E., Reiner R.C., Smith M., Feigin V.L., Vos T., Murray C.J.L., Sunshine J.E., Yost M.G., Ellenbogen R.G., Kalani R., Morrison S.D., Crowe C.S., Massenburg B.B., Theadom A., Te Ao B.J., Balalla S., Jones K.M., Ofori-Asenso R., Li S., Sobhani S., Hosseini S.M., Mansournia M.A., Yaseri M., Anjomshoa M., Mousavi S.M., Haj-Mirzaian A.A., Haj-Mirzaian A.A., Malekzadeh R., Poustchi H., Roshandel G., Sepanlou S.G., Afarideh M., Esteghamati A., Esteghamati S., Ganji M., Kasaeian A., Rahimi-Movaghar A., Eskandarieh S., Sahraian M.A., Shams-Beyranvand M., Abbasi N., Farzadfar F., Irvani S.N., Rahimi-Movaghar V., Salamati P., Sharif-Alhoseini M., Fereshtehnejad S.M., Mate K.K.V., Abdulkader R., Abraha H.N., Kassa T.D., Nirayo Y.L., Weldegwergs K.G., Gezae K.E., Zenebe Z.M., Degefa M.G., Kahsay A., Asgedom S.W., Gebre A.K., Yimer E.M., Belachew A.B., Meles H., Adsuar J.C., Zodpey S., Agrawal S., Awasthi A., Kumar G.A., Ahmadi A., Najafi F., Rajati F., Khazaie H., Farzaei M.H., Moradi M., Rezaei S., Soofi M., Siabani S., Rezaeian S., Ahmed M.B., Gebrehiwot T.T., Feyissa G.T., Hussen M.A., Aichour A., Aichour I., Aichour M.T.E., Akinyemi R.O., Owolabi M.O., Akseer N., Bhutta Z.A., Badawi A., Alahdab F., Kassa G.M., Alebel A., Wagnew F.W.S., Belay Y.A., Leshargie C.T., Alghnam S.A., Ali B.A., Alsharif U., Temsah M.H., Altirkawi K., Davitoiu D.V., Hostiuc S., Beuran M., Negoi I., Andrei C.L., Ansari H., Ansha M.G., Antonio C.A.T., Appiah S.C.Y., Levi M., Ariani F., Biffino M., Asefa N.G., Atique S., Rahman M.A., Wijeratne T., Ayala Quintanilla B.P., Ayuk T.B., Azzopardi P.S., Rafiei A., Badali H., Mohammadi M., Moosazadeh M., Daryani A., Banstola A., Tran K.B., Barker-Collo S.L., Bärnighausen T.W., Bedi N., Tehrani-Banihashemi A., Shabaninejad H., Behzadifar M.M., Kabir A., Yousefifard M., Moradi-Lakeh M., Behzadifar M.M., Bekele B., Hassen H.Y., Henok A., Biadgo B., Bennett D.A., Goulart A.C., Bensenor I.M., Lotufo P.A., Wang Y.P., Yisma E., Berhane A., Deribe K., Yasin Y.J., Demoz G.T., Bhalla A., Bhaumik S., Bijani A., Zamani M., Bililign N., Kumar M., Birungi C., Boufous S., Brazinova A., Brown A.W., Car M., Majeed A., Rawaf S., Rawaf D.L., Shoman H., Cárdenas R., Carrero J.J., El-Khatib Z., Roy N., Carvalho F., Santos J.V., Fernandes E., Silva J.P., Castañeda-Orjuela C.A., Hoffman H.J., Catalá-López F., Chaiah Y., Temsah O., Champs A.P., Chang J.C., Choi J.Y.J., Christopher D.J., Cooper C., Djalalinia S., Do H.P., Nguyen T.H., Doku D.T., Drake T.M., Sheikh A., Soyiri I.N., Dubey M., Santric Milicevic M.M.M., Dubljanin E., Faro A., Filip I., Radfar A., Fischer F., Fukumoto T., Gankpe F.G., Gopalkrishna G., Haagsma J.A., Polinder S., Khoja A.T., Pinilla-Monsalve G.D., Hamadeh R.R., Hamidi S., Haro J.M., Hassankhani H., Havmoeller R., Hegazy M.I., Hendrie D., Miller T.R., Hibstu D.T., Kassa Z.Y., Hole M.K., Homaie Rad E., Hu G., Ilesanmi O.S., Jayaraman S., Jha R.P., Jonas J.B., Moazen B., Jorjoran Shushtari Z., Jozwiak J.J., Jürisson M., Kahssay M., Liben M.L., Karch A., Kengne A.P., Khader Y.S., Safari H., Khafaie M.A., Khalid N., Khan E.A., Khan M.S., Usman M.S., Siddiqi T.J., Khang Y.H., Khubchandani J., Kiadaliri A.A., Kim D., Kim Y.E., Yoon S.J., Kisa A., Winkler A.S., Koyanagi A., Kuate Defo B., Kucuk Bicer B., Lalloo R., Moradinazar M., Lami F.H., Lansingh V.C., Laryea D.O., Latifi A., Safiri S., Lunevicius R., Mahotra N.B., Majdan M., Manda A.L., Mehndiratta M.M., Mehta V., Melese A., Memiah P.T.N., Mendoza W., Mengistu G., Shiferaw M.S., Tekle M.G., Meretoja T.J., Meretoja A., Szoeke C.E.I., Mestrovich T., Miazgowski T., Mini G.K., Mirica A., Mirrakhimov E.M., Molokhia M., Monast L., Ronfani L., Mondello S., Moradi G., Moschos M.M., Murthy S., Musa K.I., Mustafa G., Naik G., Schwebel D.C., Nangia V., Nascimento B.R., Ningrum D.N.A., Nyasulu P.S., Renzaho A.M.N., Ogbo F.A., Oh I.H., Okoro A., Olagunju A.T., Olagunju T.O., Olivares P.R., Otstavnov S.S., Dnb M.P.A., Pakhale S., Pandey A.R., Pesudovs K., Prakash S., Qorbani M., Rafay A., Rahman M.A., Stokes M.A., Shariful Islam S.M., Rai R.K., Ram U., Reis C., Resnikoff S., Roever L., Sunguy B.F., Ruhago G.M., Saddik B., Saldanha R.D.F., Samy A.M., Sanabria J., Sartorius B., Satpathy M., Schneider I.J.C., Shaikh M.A.A., Sharif M., She J., Shen J., Sheth K.N., Shibuya K., Shigematsu M., Shiri R., Shiue I., Silveira D.A., Soares Filho A.M., Sinha D.N., Soriano J.B., Stein D.J., Sufiyan M.B., Sykes B.L., Tabarés-Seisdedos R., Tortajada-Girbés M., Topor-Madry R., Tran B.X., Tudor Car L., Ukwaja K.N., Ullah I., Uthman O.A., Valdez P.R., Vasankari T.J., Venketasubramanian N., Violante F.S., Waheed Y., Werdecker A., Wyper G.M.A., Yano Y., Ye P., Yip P., Yonemoto N., Younis M.Z., Yu C., Zaidi Z., Zaman S.B. (2019). Global, regional, and national burden of traumatic brain injury and spinal cord injury, 1990–2016: A systematic analysis for the Global Burden of Disease Study 2016. Lancet Neurol..

[b0140] Jamjoom A.A.B., Rhodes J., Andrews P.J.D., Grant S.G.N. (2021). The synapse in traumatic brain injury. Brain.

[b0145] Jones S.R. (2016). When brain rhythms aren’t ‘rhythmic’: implication for their mechanisms and meaning. Curr. Opin. Neurobiol..

[b0150] Kirov I.I., Tal A., Babb J.S., Reaume J., Bushnik T., Ashman T.A., Flanagan S., Grossman R.I., Gonen O. (2013). Proton MR spectroscopy correlates diffuse axonal abnormalities with post-concussive symptoms in mild traumatic brain injury. J. Neurotrauma.

[b0155] Kopell N., Ermentrout G.B., Whittington M.A., Traub R.D. (2000). Gamma rhythms and beta rhythms have different synchronization properties. Proc. Natl. Acad. Sci. U. S. A..

[b0160] Lewine J.D., Davis J.T., Sloan J.H., Kodituwakku P.W., Orrison W.W. (1999). Neuromagnetic assessment of pathophysiologic brain activity induced by minor head trauma. AJNR. Am. J. Neuroradiol..

[b0165] Little S., Bonaiuto J., Barnes G., Bestmann S. (2019). Human motor cortical beta bursts relate to movement planning and response errors. PLoS Biol..

[b0170] MacFarlane M.P., Glenn T.C. (2015). Neurochemical cascade of concussion. Brain Inj.

[b0175] Marklund N., Vedung F., Lubberink M., Tegner Y., Johansson J., Blennow K., Zetterberg H., Fahlström M., Haller S., Stenson S., Larsson E.-M., Wall A., Antoni G. (2021). Tau aggregation and increased neuroinflammation in athletes after sports-related concussions and in traumatic brain injury patients-a PET/MR study. NeuroImage Clin..

[b0180] McColgan P., Joubert J., Tabrizi S.J., Rees G. (2020). The human motor cortex microcircuit: insights for neurodegenerative disease. Nat. Rev. Neurosci..

[b0185] McCrory P., Meeuwisse W., Dvořák J., Aubry M., Bailes J., Broglio S., Cantu R.C., Cassidy D., Echemendia R.J., Castellani R.J., Davis G.A., Ellenbogen R., Emery C., Engebretsen L., Feddermann-Demont N., Giza C.C., Guskiewicz K.M., Herring S., Iverson G.L., Johnston K.M., Kissick J., Kutcher J., Leddy J.J., Maddocks D., Makdissi M., Manley G.T., McCrea M., Meehan W.P., Nagahiro S., Patricios J., Putukian M., Schneider K.J., Sills A., Tator C.H., Turner M., Vos P.E. (2017). Consensus statement on concussion in sport—the 5th international conference on concussion in sport held in Berlin, October 2016. Br. J. Sports Med..

[b0190] McCrory, P., Meeuwisse, W., Johnston, K., Dvorak, J., Aubry, M., Molloy, M., Cantu, R., 2009. Consensus statement on concussion in sport – The 3rd International Conference on concussion in sport, held in Zurich, November 2008. J. Clin. Neurosci. 16, 755–763. 10.1016/J.JOCN.2009.02.002. 10.1016/j.jocn.2009.02.002 19410148

[b0195] Nolte G. (2003). The magnetic lead field theorem in the quasi-static approximation and its use for magnetoenchephalography forward calculation in realistic volume conductors. Phys. Med. Biol..

[b0200] O’Neill G.C., Barratt E.L., Hunt B.A.E., Tewarie P.K., Brookes M.J. (2015). Measuring electrophysiological connectivity by power envelope correlation: A technical review on MEG methods. Phys. Med. Biol..

[b0205] O’Neill G.C., Bauer M., Woolrich M.W., Morris P.G., Barnes G.R., Brookes M.J. (2015). Dynamic recruitment of resting state sub-networks. Neuroimage.

[b0210] Oh A., Vidal J., Taylor M.J., Pang E.W. (2014). Neuromagnetic correlates of intra- and extra-dimensional set-shifting. Brain Cogn..

[b0215] Oostenveld R., Fries P., Maris E., Schoffelen J.M. (2011). FieldTrip: Open source software for advanced analysis of MEG, EEG, and invasive electrophysiological data. Comput. Intell. Neurosci..

[b0220] Pakenham D.O., Quinn A.J., Fry A., Francis S.T., Woolrich M.W., Brookes M.J., Mullinger K.J. (2020). Post-stimulus beta responses are modulated by task duration. Neuroimage.

[b0225] Pearce A.J., Rist B., Fraser C.L., Cohen A., Maller J.J. (2018). Neurophysiological and cognitive impairment following repeated sports concussion injuries in retired professional rugby league players. Brain Inj..

[b0230] Pfurtscheller G., Stancák A., Neuper C. (1996). Post-movement beta synchronization. A correlate of an idling motor area?. Electroencephalogr. Clin. Neurophysiol..

[b0235] Proskovec A.L., Shah B.R., Yu F.F., Achilleos M., Maldjian J.A., Davenport E.M. (2020). Magnetoencephalography and Mild Traumatic Brain Injury. Adv. Clin. Radiol..

[b0240] Seedat Z.A., Quinn A.J., Vidaurre D., Liuzzi L., Gascoyne L.E., Hunt B.A.E., O’Neill G.C., Pakenham D.O., Mullinger K.J., Morris P.G., Woolrich M.W., Brookes M.J. (2020). The role of transient spectral ‘bursts’ in functional connectivity: A magnetoencephalography study. Neuroimage.

[b0245] Sekihara K., Nagarajan S.S., Poeppel D., Marantz A. (2004). Asymptotic SNR of scalar and vector minimum-variance beanformers for neuromagnetic source reconstruction. IEEE Trans. Biomed. Eng..

[b0250] Sherman M.A., Lee S., Law R., Haegens S., Thorn C.A., Hämäläinen M.S., Moore C.I., Jones S.R. (2016). Neural mechanisms of transient neocortical beta rhythms: Converging evidence from humans, computational modeling, monkeys, and mice. Proc. Natl. Acad. Sci. U. S. A..

[b0255] Stoffers D., Bosboom J.L.W., Deijen J.B., Wolters E.C., Berendse H.W., Stam C.J. (2007). Slowing of oscillatory brain activity is a stable characteristic of Parkinson’s disease without dementia. Brain.

[b0260] Tewarie P., Hunt B.A.E., O’Neill G.C., Byrne A., Aquino K., Bauer M., Mullinger K.J., Coombes S., Brookes M.J. (2019). Relationships Between Neuronal Oscillatory Amplitude and Dynamic Functional Connectivity. Cereb. Cortex.

[b0265] Tzourio-Mazoyer N., Landeau B., Papathanassiou D., Crivello F., Etard O., Delcroix N., Mazoyer B., Joliot M. (2002). Automated anatomical labeling of activations in SPM using a macroscopic anatomical parcellation of the MNI MRI single-subject brain. Neuroimage.

[b0270] van Ede F., Quinn A.J., Woolrich M.W., Nobre A.C. (2018). Neural Oscillations: Sustained Rhythms or Transient Burst-Events?. Trends Neurosci.

[b0275] Van Veen B.D., van Drongelen W., Yuchtman M., Suzuki A. (1997). Localization of brain electrical activity via linearly constrained minimum variance spatial filtering. IEEE Trans. Biomed. Eng..

[b0280] Vidaurre D., Hunt L.T., Quinn A.J., Hunt B.A.E., Brookes M.J., Nobre A.C., Woolrich M.W. (2018). Spontaneous cortical activity transiently organises into frequency specific phase-coupling networks. Nat. Commun..

[b0285] Whitlock J.R. (2017). Posterior parietal cortex. Curr. Biol..

[b0290] Zhang J., Hadj-Moussa H., Storey K.B. (2017). Current Progress of High-Throughput MicroRNA Differential Expression Analysis and Random Forest Gene Selection for Model and Non-Model Systems: an R Implementation. J. Integr. Bioinform..

[b0295] Zhang J., Hadj-Moussa H., Storey K.B. (2016). Current progress of high-throughput microRNA differential expression analysis and random forest gene selection for model and non-model systems: an R implementation. J. Integr. Bioinform..

[b0300] Zhang J., Safar K., Emami Z., Ibrahim G.M., Scratch S.E., da Costa L., Dunkley B.T. (2020). Local and large-scale beta oscillatory dysfunction in males with mild traumatic brain injury. J. Neurophysiol..

[b0305] Zich C., Quinn A.J., Mardell L.C., Ward N.S., Bestmann S. (2020). Dissecting Transient Burst Events. Trends Cogn. Sci..

