# Supplementary material for: Mild traumatic brain injury impairs the coordination of intrinsic and motor-related neural dynamics
Source: Neuroimage Clin. 2021 Oct 1;32:102841. doi: 10.1016/j.nicl.2021.102841 (PMC8517919; doi:10.1016/j.nicl.2021.102841)
Supplement: Supplementary data 1 [file mmc1.pdf]

Supplementary materials

1. MRI acquisition parameters for mTBI group:

Anatomical T1-weighted images acquired on 3T Magnetom Tim Trio (Siemens AG, Erlangen, Germany) MRI system located at The Hospital for Sick Children in Toronto, using a 12-channel head coil running an MPRAGE pulse sequence (TR = 2,300 ms; TE = 2.9 ms; flip angle = 9◦; field of view = 240 × 256 × 192 mm; slice thickness 1 mm3).

2. Scatter plot for global burst connectivity vs symptom severity:

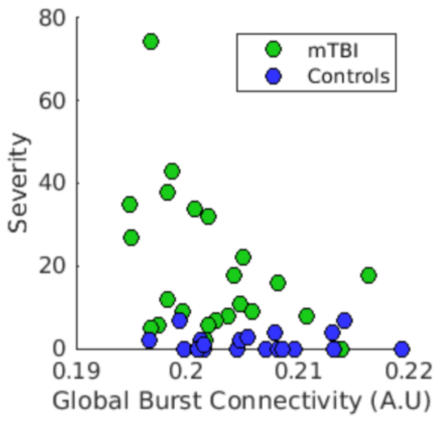

3. Scatter plot for burst connectivity between the motor cortices during the Post Movement Beta Rebound vs Symptom severity:

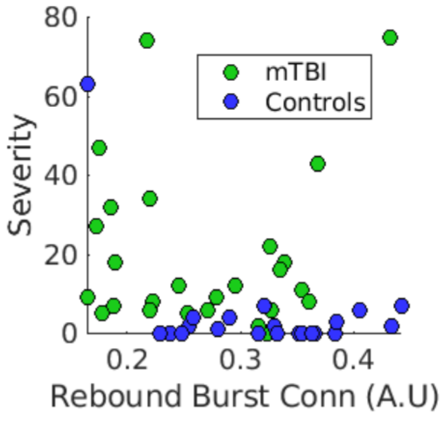

Note that one control subject reported much more severe symptoms than the average. Given that the exclusion criteria for controls did not require presentation without symptoms we did not exclude this subject from analysis.

4. Supplementary video description:

Video showing the time evolution of burst coincidence connectivity during a single trial relative to a button press. The top three rows of panels contain glass brain plots showing the connection strengths as measured via the Jaccard index for the mTBI group (first row),

the control group (second row) and the difference between groups (third row). The bottom panel shows traces for the global average connectivity for both groups. The vertical line in the bottom panel indicates the time point within the trial for which the glass brains are plotted.
